# Author Correction: CryoEM structural exploration of catalytically active enzyme pyruvate carboxylase

**DOI:** 10.1038/s41467-022-34543-8

**Published:** 2022-11-16

**Authors:** Jorge Pedro López-Alonso, Melisa Lázaro, David Gil-Cartón, Philip H. Choi, Alexandra Dodu, Liang Tong, Mikel Valle

**Affiliations:** 1 CIC bioGUNE, Basque Research & Technology Alliance (BRTA), Bizkaia Technology Park, Derio, Bizkaia Spain; 2 IKERBASQUE, Basque Foundation for Science, Bilbao, Spain; 3 Department of Biological Sciences, Columbia University, New York, NY USA; 4 Present Address: Basque Resource for Electron Microscopy, Instituto Biofisika (CSIC - UPV/EHU), Leioa, Spain

**Keywords:** Cryoelectron microscopy, Transferases, Enzyme mechanisms

Correction to: *Nature Communications* 10.1038/s41467-022-33987-2, published online 19 October 2022

The original version of this article omitted from the author list the 5th author Alexandra Dodu, who is from the ‘CIC bioGUNE’. Additionally, the following was added to the Author Contributions: ‘J.P.L., M.L., A.D. and P.H.C. produced protein samples and performed the biochemical assays.’ This has been corrected in both the PDF and HTML versions of the article.

