# Supplementary material for: CryoEM structural exploration of catalytically active enzyme pyruvate carboxylase
Source: Nat Commun. 2022 Oct 19;13:6185. doi: 10.1038/s41467-022-33987-2 (PMC9581989; doi:10.1038/s41467-022-33987-2)
Supplement: Supplementary file 1 — Supplementary information [file 41467_2022_33987_MOESM1_ESM.pdf]

Supplementary Information

Supplementary Figures

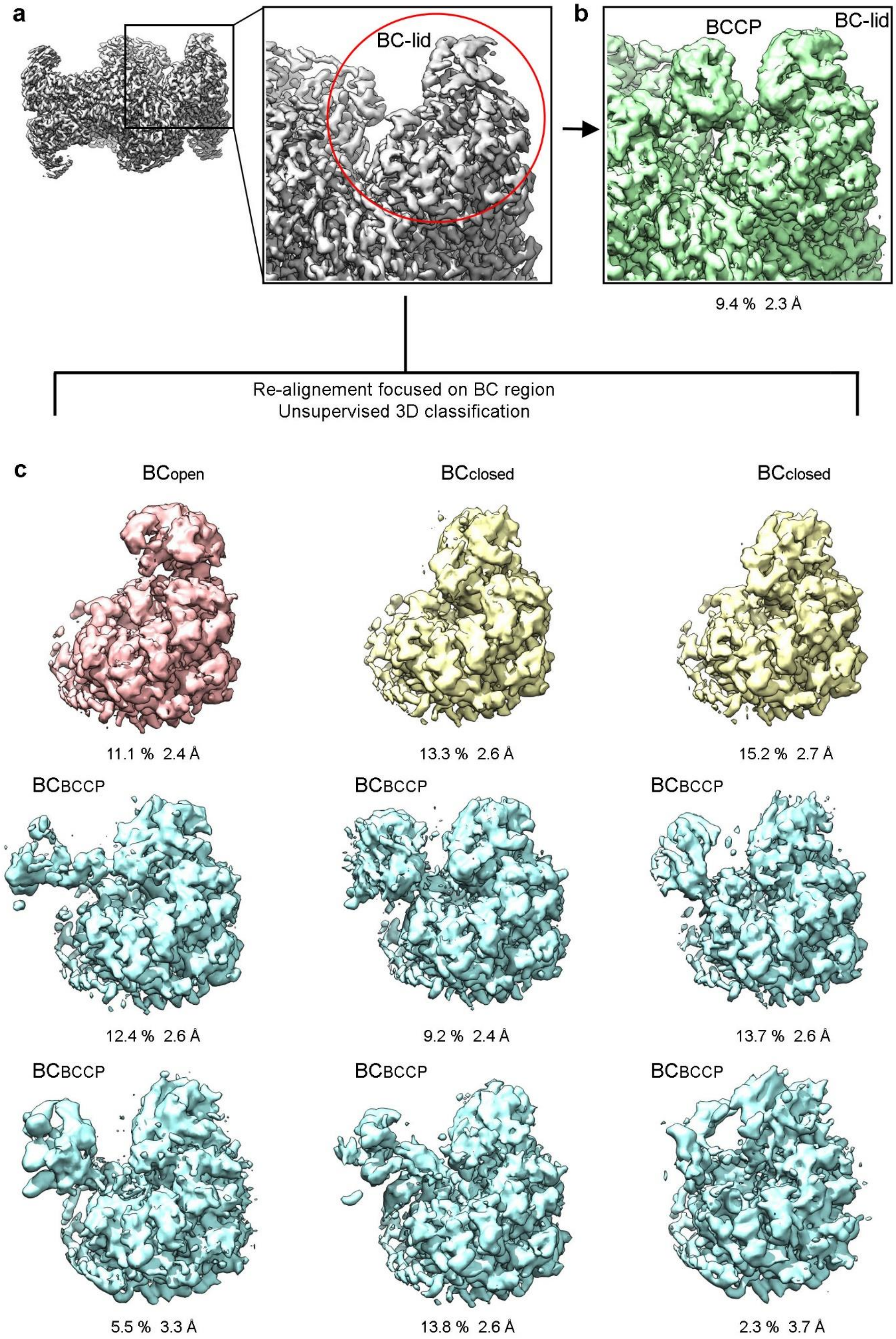

Supplementary Fig. 1. Classification at the BC reaction center.

a CryoEM map for LlPC in the presence of acetyl-CoA, substrates and co-factors as shown in figure 1b. The red circle in the inset shows the region at the BC site used for the masked focused classification. **b** Detail of the cryoEM map of the BC_{react} class isolated after the classification. **c** Classes obtained after re-alignment of the BC region and focused classification. The percentage of particles and the estimated resolutions after refinement are indicated.

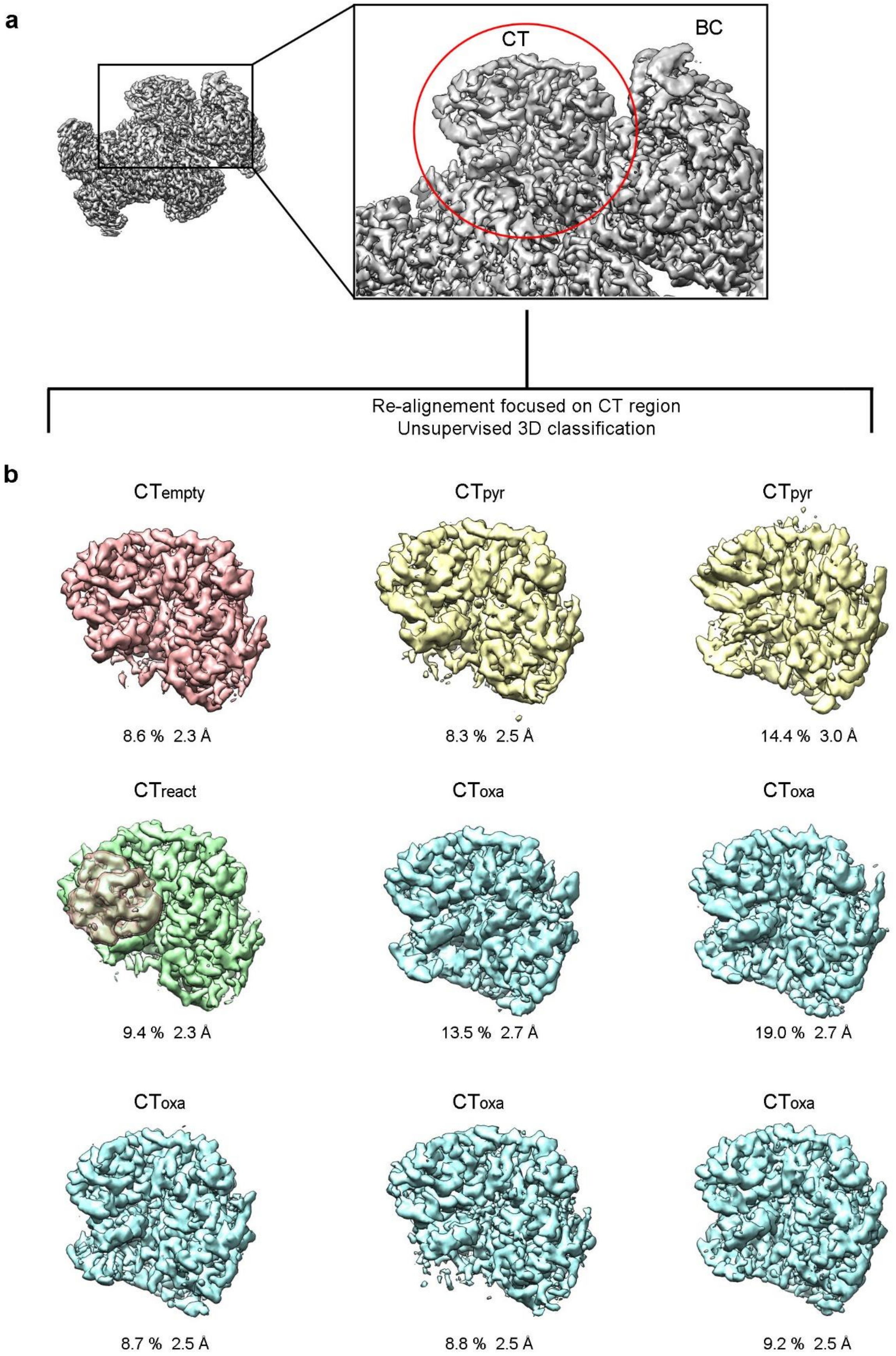
a
CT
BC
Re-alignement focused on CT region
Unsupervised 3D classification
b
CTempty
CTpyr
CTpyr
8.6 % 2.3 Å
8.3 % 2.5 Å
14.4 % 3.0 Å
CTreact
CToxa
CToxa
9.4 % 2.3 Å
13.5 % 2.7 Å
19.0 % 2.7 Å
CToxa
CToxa
CToxa
8.7 % 2.5 Å
8.8 % 2.5 Å
9.2 % 2.5 Å

Supplementary Fig. 2. Classification at the CT reaction center.

a CryoEM map for LlPC in the presence of acetyl-CoA, substrates and co-factors as shown in figure 1b. The red circle in the inset shows the region at the CT site used for re-alignment of the CT region and a masked focused classification. **b** Classes obtained after re-alignment of the CT region and focused classification. The percentage of particles and the estimated resolutions after refinement are indicated.

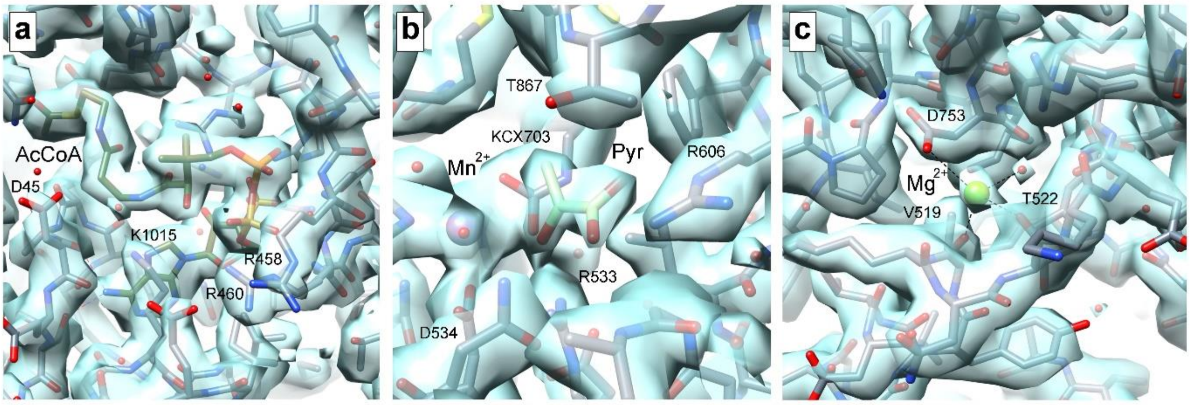

Supplementary Fig. 3. Structural details of the density map and the atomic model for LlPC.

The three panels show different regions of the cryoEM map and the derived atomic model to illustrate the achieved resolution. **a** Binding site for acetyl-CoA (CoA) at the PT domain. **b** Pyruvate binding site at the CT domain of the CT_{pyr} map. **c** Region connecting the CT and PT domains with an additional Mg^{2+} ion.

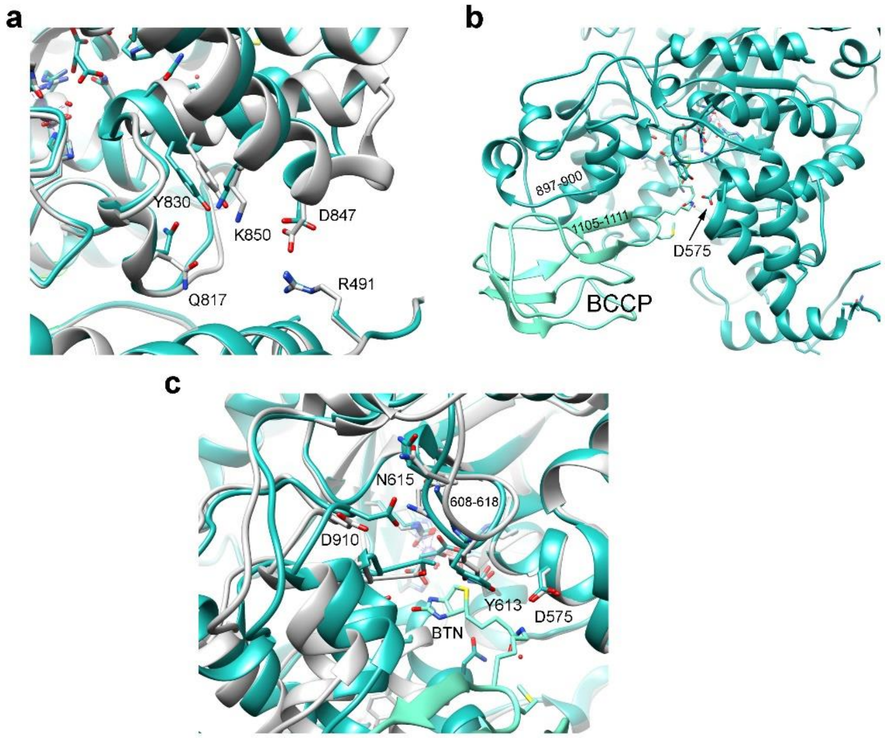

Supplementary Fig. 4. Overlap of CT_{react} (cyan) and CT_{empty} (grey).

a Interface between the funnel subdomain with CT in the other layer. **b** Binding of BCCP to CT. BCCP is colored light cyan. Many interactions are established between the loop 897-900 in the funnel domain with the strand 1105-1111 in the BCCP. **c** The interaction between Asp910 and the main chain of Asn615 in CT_{react} modifies the position of the loop 608-618 resulting in a new interaction of Tyr613 with Asp575 allowing the biotin to enter to the active site.

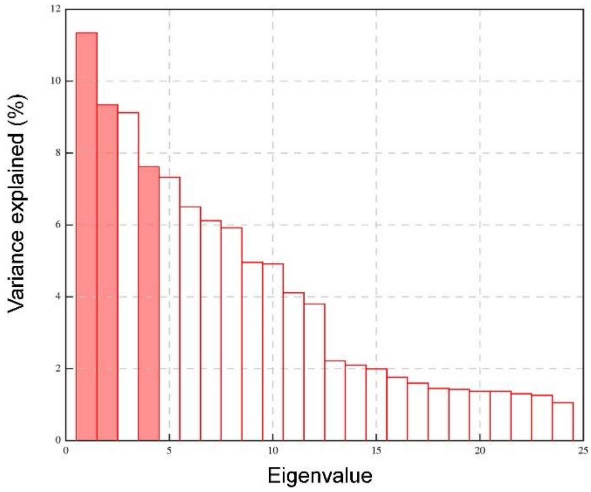

Supplementary Fig. 5. Percentage of variance explained by each eigenvalue during multibody analysis
of LlPC.

Eigenvalues for vectors 1, 2, and 4 shown in figure 7 are in red bars. Source data are provided as a Source Data file.

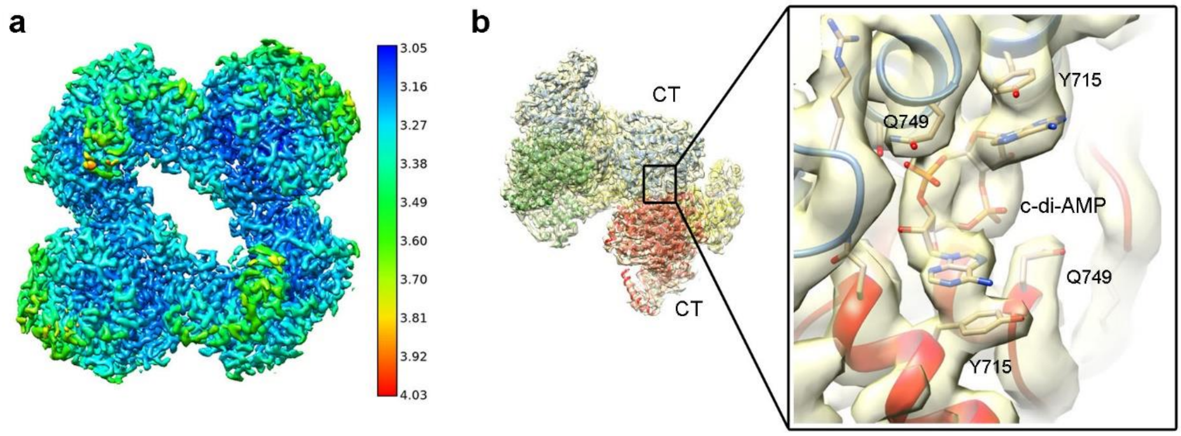

Supplementary Fig. 6. CryoEM map and atomic model for LlPC in the presence of c-di-AMP and acetyl-CoA.

a CryoEM map for LlPC in the presence of c-di-AMP and acetyl-CoA. The 3D map is colored by estimates of local resolution, being the overall resolution 3.37 Å. **b** Structural details of the interaction of c-di-AMP at its binding site in the boundary between CT domains.

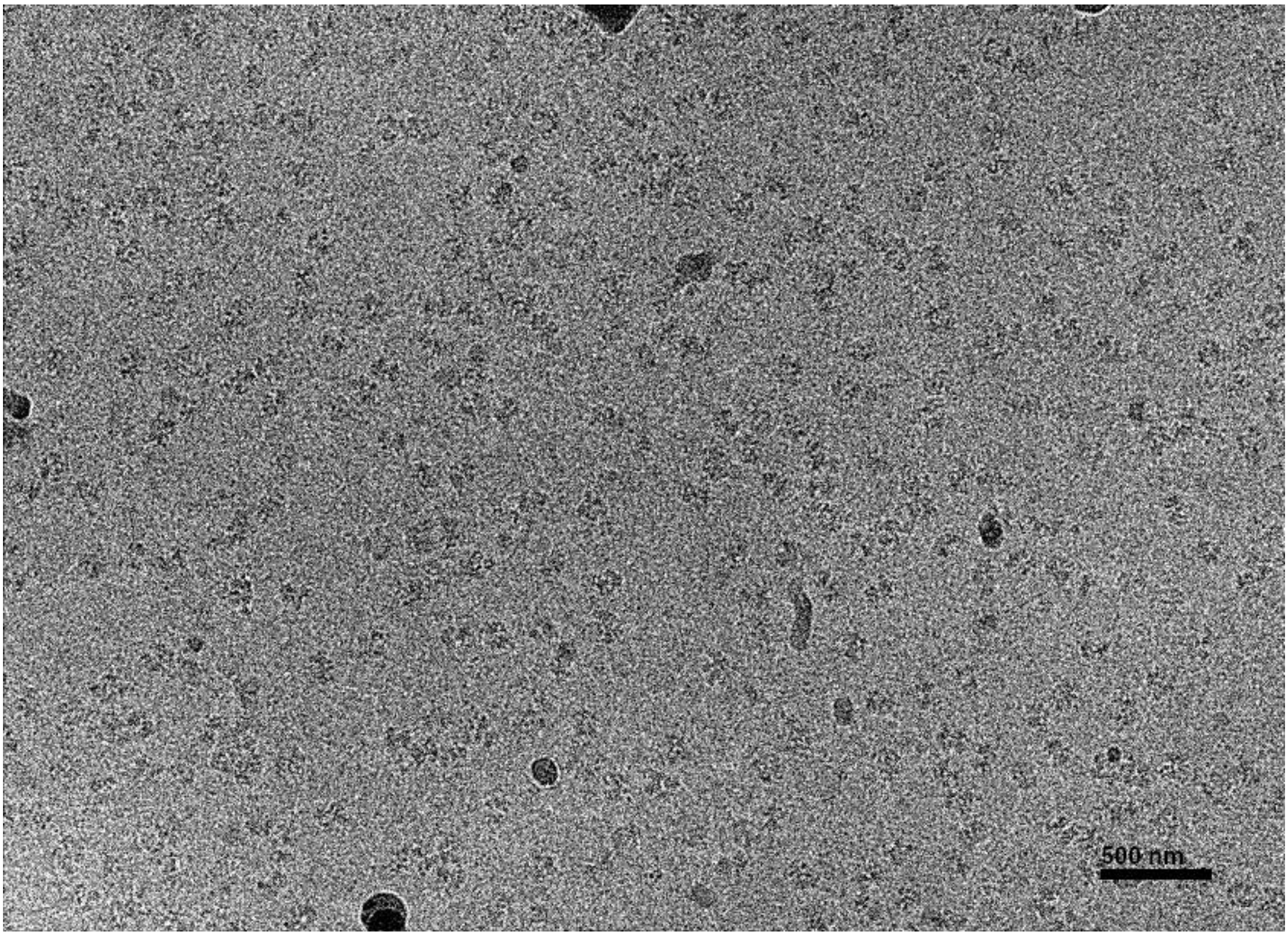

Supplementary Fig. 7. Representative cryoEM micrograph.

Representative cryoEM micrograph for the LlPC sample in the presence of substrates and co-fa ctors and acetyl-CoA.
