# Supplementary material for: CryoEM structural exploration of catalytically active enzyme pyruvate carboxylase
Source: Nat Commun. 2022 Oct 19;13:6185. doi: 10.1038/s41467-022-33987-2 (PMC9581989; doi:10.1038/s41467-022-33987-2)
Supplement: Supplementary file 3 — Reporting Summary [file 41467_2022_33987_MOESM3_ESM.pdf]

nature portfolio

Corresponding author(s): Mikel Valle

Last updated by author(s): Sep 23, 2022

Reporting Summary

Nature Portfolio wishes to improve the reproducibility of the work that we publish. This form provides structure for consistency and transparency in reporting. For further information on Nature Portfolio policies, see our Editorial Policies and the Editorial Policy Checklist.

Statistics

For all statistical analyses, confirm that the following items are present in the figure legend, table legend, main text, or Methods section.

n/a	Confirmed	
☐	☒	The exact sample size (*n*) for each experimental group/condition, given as a discrete number and unit of measurement
☒	☐	A statement on whether measurements were taken from distinct samples or whether the same sample was measured repeatedly
☒	☐	The statistical test(s) used AND whether they are one- or two-sided<br>*Only common tests should be described solely by name; describe more complex techniques in the Methods section.*
☒	☐	A description of all covariates tested
☒	☐	A description of any assumptions or corrections, such as tests of normality and adjustment for multiple comparisons
☒	☐	A full description of the statistical parameters including central tendency (e.g. means) or other basic estimates (e.g. regression coefficient) AND variation (e.g. standard deviation) or associated estimates of uncertainty (e.g. confidence intervals)
☒	☐	For null hypothesis testing, the test statistic (e.g. *F*, *t*, *r*) with confidence intervals, effect sizes, degrees of freedom and *P* value noted<br>*Give P values as exact values whenever suitable.*
☒	☐	For Bayesian analysis, information on the choice of priors and Markov chain Monte Carlo settings
☒	☐	For hierarchical and complex designs, identification of the appropriate level for tests and full reporting of outcomes
☒	☐	Estimates of effect sizes (e.g. Cohen's *d*, Pearson's *r*), indicating how they were calculated

Our web collection on statistics for biologists contains articles on many of the points above.

Software and code

Policy information about availability of computer code

Data collection	EPU 2.4.0.54 from ThermoFisher
Data analysis	MotionCor2 1.2.6; CTFfind 4.1.5; Xmipp 2.5; Scipion 1.1; Relion 3.0.6 and Relion 3.1.0; LocScale 1.0, Phenix 1.20.1; Coot 0.9.1; Chimera 1.10.2

For manuscripts utilizing custom algorithms or software that are central to the research but not yet described in published literature, software must be made available to editors and reviewers. We strongly encourage code deposition in a community repository (e.g. GitHub). See the Nature Portfolio guidelines for submitting code & software for further information.

Data

Policy information about availability of data

All manuscripts must include a data availability statement. This statement should provide the following information, where applicable:

- Accession codes, unique identifiers, or web links for publicly available datasets
- A description of any restrictions on data availability
- For clinical datasets or third party data, please ensure that the statement adheres to our policy

CryoEM maps have been deposited in the Electron Microscopy Data Bank (https://www.ebi.ac.uk/emdb/) with IDs: EMD-15028 (LIPC overall map); EMD-15033 (BCreact); EMD-15035 (BCopen); EMD-15034 (BCclosed); EMD-15030 (CTempty); EMD-15032 (CTpyr); EMD-15031 (CTreact); EMD-15029 (CToxa); and EMD-15037 (LIPC with acetyl CoA and c-di-AMP).

Generated atomic models have been deposited in the Protein Data Bank (https://www.rcsb.org/) with codes: 7ZYY (LlPC overall map); 7ZZ3 (BCreact); 7ZZ5 (BCopen); 7ZZ4 (BCclosed); 7ZZ0 (CTempty); 7ZZ2 (CTpyr); 7ZZ1 (CTreact); 7ZYZ (CToxa); and 7ZZ8 (LlPC with acetyl CoA and c-di-AMP).

Human research participants

Policy information about studies involving human research participants and Sex and Gender in Research.

Reporting on sex and gender	This study does not involve human research.
Population characteristics	See above
Recruitment	See above
Ethics oversight	See above

Note that full information on the approval of the study protocol must also be provided in the manuscript.

Field-specific reporting

Please select the one below that is the best fit for your research. If you are not sure, read the appropriate sections before making your selection.

[x] Life sciences [ ] Behavioural & social sciences [ ] Ecological, evolutionary & environmental sciences

For a reference copy of the document with all sections, see nature.com/documents/nr-reporting-summary-flat.pdf

Life sciences study design

All studies must disclose on these points even when the disclosure is negative.

Sample size	The initial sample size was set by automatic selection using Laplacian autopick in Relion 3.06. This initial set of single particles (4,578,464) must be cleaned to select real particles that represent the sample.
Data exclusions	Several rounds of reference-free 2D class averaging and unsupervised 3D classification were used to clean the data until a final number of 342,273 particles. In the 2D classification, averages showing high resolution information were selected. In the 3D classification, cryoEM maps displaying undamaged sample were selected. The rest of the images were removed from the analysis.
Replication	We have not attempted to reproduce the study. It would be overwellming to do the entire analysis again.
Randomization	There is only one group in the samples and no randomization is used.
Blinding	Since there is not randomization of samples in different groups there is no blinding.

Reporting for specific materials, systems and methods

We require information from authors about some types of materials, experimental systems and methods used in many studies. Here, indicate whether each material, system or method listed is relevant to your study. If you are not sure if a list item applies to your research, read the appropriate section before selecting a response.

Materials & experimental systems

n/a	Involved in the study	
[x]	[ ]	Antibodies
[x]	[ ]	Eukaryotic cell lines
[x]	[ ]	Palaeontology and archaeology
[x]	[ ]	Animals and other organisms
[x]	[ ]	Clinical data
[x]	[ ]	Dual use research of concern

Methods

n/a	Involved in the study	
[x]	[ ]	ChIP-seq
[x]	[ ]	Flow cytometry
[x]	[ ]	MRI-based neuroimaging
