# Supplementary material for: CryoEM structural exploration of catalytically active enzyme pyruvate carboxylase
Source: Nat Commun. 2022 Oct 19;13:6185. doi: 10.1038/s41467-022-33987-2 (PMC9581989; doi:10.1038/s41467-022-33987-2)
Supplement: Supplementary file 4 — Description of Additional Supplementary Files [file 41467_2022_33987_MOESM4_ESM.pdf]

Description of Additional Supplementary Files:

Suppl. Movie M1: Morphing between the atomic structures modeled for BCopen and BCclosed states. Sub-domains are colored as in figure 4.

Suppl. Movie M2: Morphing between the atomic structures modeled for CTempty, CTpyr, CTreact, and CToxa classes. Residue Arg606 is shown to illustrate its transition. Sub-domains are colored as in figure 6.
